# Establishing a signature based on immunogenic cell death-related gene pairs to predict immunotherapy and survival outcomes of patients with hepatocellular carcinoma

**DOI:** 10.18632/aging.204419

**Published:** 2022-12-14

**Authors:** Jianying Ma, Lianghong Kuang, Rong Zhao

**Affiliations:** 1 Department of Breast Surgery, Thyroid Surgery, Huangshi Central Hospital, Affiliated Hospital of Hubei Polytechnic University, Edong Healthcare Group, Huangshi, Hubei 435000, People’s Republic of China; 2 Department of Neurology, Huangshi Central Hospital, Affiliated Hospital of Hubei Polytechnic University, Edong Healthcare Group, Huangshi, Hubei 435000, People’s Republic of China; 3 Department of Anesthesiology, Huangshi Central Hospital, Affiliated Hospital of Hubei Polytechnic University, Edong Healthcare Group, Huangshi, Hubei 435000, People’s Republic of China

**Keywords:** immunogenic cell death, hepatocellular carcinoma, prognosis, tumor microenvironment, immunotherapy

## Abstract

Immunogenic cell death (ICD) is a type of regulated cell death (RCD) triggered by various stresses that are involved in activating the immune system against cancer in immunocompetent hosts. However, no previous study has investigated the regulation of ICD-related gene pairs involved in hepatocellular carcinoma (HCC). A prognostic signature composed of 8 ICD-related gene pairs was generated that was capable of reliably separating patients with HCC into low- and high-risk subgroups with differing overall survival rates. Significant correlations were observed between risk score and surgical procedure, vascular tumor cell type, recurrence status, tumor status, and stages. The risk score was confirmed to be an independent prognostic factor for HCC and subsequently was employed to construct a prognostic nomogram. Low-risk patients were characterized by higher levels of immune cell infiltration, lower stromal and immune scores, higher tumor purity, higher expression of most immune checkpoints, and higher tumor mutational burden (TMB), revealing different levels of immunological functional pathways between different risk HCC patient cohorts. Furthermore, immunophenoscore (IPS) and Tumor Immune Dysfunction and Exclusion (TIDE) scores demonstrated that patients in the low-risk group are more likely to be sensitive to immunotherapy. In conclusion, the signature conducted by ICD-related gene pairs is a promising biomarker for the prediction of HCC patient outcomes and immunotherapeutic responses.

## INTRODUCTION

Hepatocellular carcinoma (HCC) is a deadly disease and the most frequent malignant neoplasm of the liver in the world. There are an estimated 905,677 new cases and 830,180 deaths of liver cancer around the world in 2020 [1]. At present, treatments for HCC include hepatectomy, transarterial chemoembolization, immunotherapy, targeted therapies, and liver transplantation. Although therapeutic advances have been made, the five-year survival rate of HCC remains at 2.4% for patients with distant metastases [2]. Notably, emerging cancer immunotherapies have yielded some encouraging results based on immune checkpoint inhibitors (ICIs) [3, 4]. Unfortunately, only one-third of HCC patients respond to immunotherapies due to individual differences within populations [5]. These grim data highlight the urgent need to identify biomarkers that can predict response to immunotherapy and provide an accurate prognosis for HCC.

Immunogenic cell death (ICD) is a form of regulatory cell death (RCD) that is sufficient to activate adaptive immunity [6, 7]. It can be induced by different stimulatory and anticancer treatment modalities, including chemotherapy, targeted drugs, oncolytic viruses, physical chemotherapy, and radiation therapy [8, 9]. ICD involves the exposure and release of damage-associated molecular patterns (DAMPs) from dying tumor cells that are recognized by innate pattern recognition receptors that activate tumor-specific immune responses to directly kill cancer cells and anti-tumor immunity by binding to stimulate long-term efficacy of anticancer drugs [6, 8]. At the same time, immature dendritic cells (DCs) can be transformed into mature phenotypes, thereby accelerating the phagocytosis of antigenic components in DCs. Thus, through antigen presentation, DCs can stimulate specific T-cell responses to kill more tumor cells [10]. DAMPs mainly include surface-exposed calreticulin and heat shock protein and secreted HMGB1, ATP, ANXA1, and type I interferons [7, 9]. ICD is considered one of the most promising approaches to achieving the complete elimination of tumor cells. Although several ICD-related models have been constructed, the available evidence for their use in clinical practice is not convincing [11]. Therefore, it is of great significance to screen biomarkers that classify patients based on their response to ICD immunotherapy. The purpose of this study is to use ICD-related gene pairs to establish a novel signature that may serve as a predictor for prognosis and immunotherapeutic response in HCC patients.

## MATERIALS AND METHODS

### Sample data collection and identification of immunogenic cell death (ICD)-related genes

RNA sequencing results, clinical features of HCC patients, and normal liver specimens were available through The Cancer Genome Atlas (TCGA) project and Genotype-Tissue Expression (GTEx) database. After excluding patients with no clinical data, repeated data, incomplete clinical data, and a follow-up time of 0 days, 374 HCC patients and 160 normal samples were finally obtained. For the validation set, the GSE14520 dataset was downloaded from Gene Expression Omnibus (GEO) database. Additionally, 34 ICD-related genes were obtained from previous articles [12], and are listed in Supplementary Table 1. The distribution of clinical features of HCC cohorts were detailed in Table 1.

**Table 1:** Clinicopathologic characteristics of HCC patients in TCGA and GEO cohorts.

**Variables**	**TCGA cohort**	**GSE14520**
	**(*n* = 374)**	**(*n* = 221)**
	***N* (%)**	***N* (%)**
Age (M ± SD, years)	59.48 ± 13.45	50.75 ± 10.61
Gender		
Female	121 (32.4)	29 (13.1)
Male	253 (67.6)	192 (86.9)
Grade		161 (58.9)
1 and 2	233 (62.3)	/
3 and 4	136 (36.4)	/
Unknown	5 (1.3)	/
Tumor status		
Tumor free	162 (43.3)	/
With tumor	124 (33.2)	/
Unknown	88 (23.5)	/
Stage		
I–II	260 (69.5)	170 (76.9)
III–IV	90 (24.1)	50 (22.6)
Unknown	24 (6.4)	/
Vascular tumor cell type		
Macro	16 (4.3)	/
Micro	94 (25.1)	/
None	208 (55.6)	/
Unknown	56 (15.0)	/
Recurrence		
No	151 (40.4)	96 (43.4)
Yes	162 (43.3)	125 (56.6)
Unknown	61 (16.3)	/

### Identification of ICD-related gene pairs

Pairs of ICD-related genes were screened by cyclically singly paired. If the expression level of ICD-related gene A is greater than that of ICD-related gene B, it is recorded as 1; otherwise, it is recorded as 0, and the 0-or-1 matrixes are established. We consider that there was no relationship between pairs and prognoses if ICD-related gene pair = 0 or 1 because the patient’s survival outcome cannot be correctly predicted due to pairs without a certain rank. When the number of pairs equal to 1 or 0 was >20% and <80% of the total number of pairs, the match was deemed valid.

### Development and verification of ICD-related prognostic signature

Univariate cox regression was employed to retrieve OS-associated gene pairs. These gene pairs were then used to develop an ICD-related gene pair signature capable of predicting HCC patient prognosis through a LASSO regression method. The formula developed based on this analysis was then established as follows:

Risk score = regression coefficient (genei) × expression value (genei).

By setting the median value of the risk score calculated in the training set as the threshold, patients in the training and validation sets (GSE14520) were stratified into two risk subgroups. Survival analysis was conducted to analyze differences in survival between two risk HCC patients. The subsequent receiver operator characteristic (ROC) analysis and C-index were employed to assess the prognostic accuracy of the developed signature.

### Construction of a nomogram

To further confirm the applicability of the signature, we investigated the association between the risk score and clinical data, including age, sex, grade, surgical procedure, recurrence status, vascular tumor cell type, pathologic stage, and tumor status. The prognostic utility of the ICD-related signature was also examined through univariate analyses in both sets, and its ability to independently predict patient prognosis was examined via a multivariate approach. A nomogram including the risk score and various clinical traits (surgery procedure and stage) was constructed using the “regplot” package. The predictive capability of the nomogram was evaluated by AUC values, calibration plots, and decision-curve analysis (DCA). The area under the curve (AUC) of the ROC was used to assess the accuracy of the nomogram in 3- and 5-year survival predictions and the predictive prognostic performance between risk score and TNM stage were compared.

### Assessment of immune landscape

The interplay between the risk scores and the immune microenvironment in HCC patient tumors was assessed via the ESTIMATE algorithm, which examined the predicted stromal and immune cell content in these tumor samples. Calculations of immune infiltration statuses among the HCC patients were conducted using by CIBERSORT algorithm. A Spearman correlation analysis was conducted to investigate the correlations between risk score and the infiltration of immune cells by the CIBERSORT algorithm.

### Immunotherapeutic response analyses and drug efficacy assessment

To investigate the predictive ability of risk scores in the benefit of immunotherapeutic treatment, we first the differences in the expression of immune checkpoints between the two risk subgroups using “ggpubr” R package. Secondly, the TIDE method was utilized to predict cancer immunotherapy response and tumor immune escape in low- and high-risk populations [13]. Moreover, we analyzed the immunophenoscore (IPS) of HCC samples in the TCIA database. The correlations between tumor mutation burden (TMB) and risk score were analyzed using “ggExtra”, “ggplot2” and “ggpubr” packages in R software. The optimal cutoff value of TMB in survival data was identified through the function surv_cutpoint of the “survminer” R package, and then patients were stratified into low- and high-TMB subgroups. The OS of CRC samples between two subgroups was compared by the Kaplan-Meier method. Next, a combined survival analysis was performed for TMB and risk score. To evaluate the response of the two risk groups to different drugs, the R package “pRRophetic” was employed to evaluate the half inhibitory concentration (IC50) of some common chemotherapeutic drugs in every HCC specimen.

### Functional enrichment analysis

To understand the biological functions of risk-related differential genes and the potential signaling enrichment pathways, the functional enrichment analyses were conducted with the package “clusterProfiler”, and presented as bar graphs and bubble plots, respectively.

### Statistical analysis

R (v4.0.3; http://www.Rproject.org) was used for statistical comparisons. *P*-value < 0.05 would be considered statistically significant.

### Availability of data and materials

Publicly available datasets were analyzed in this study. This data can be found at TCGA (https://portal.gdc.cancer.gov/) and GEO (https://www.ncbi.nlm.nih.gov/geo/).

## RESULTS

### Identification of ICD-related gene pairs and establishment of the risk model in HCC

**Figure 1:**
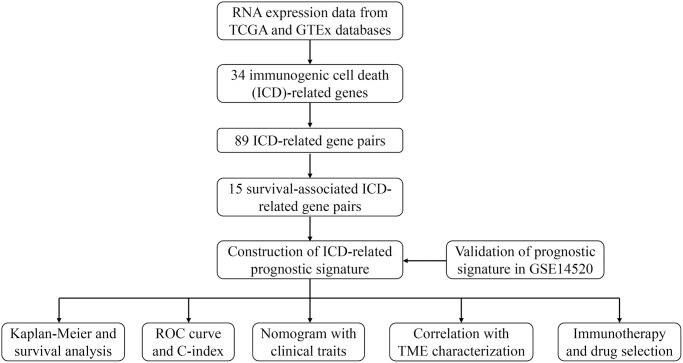
The flow chart of this study.

**Figure 2:**
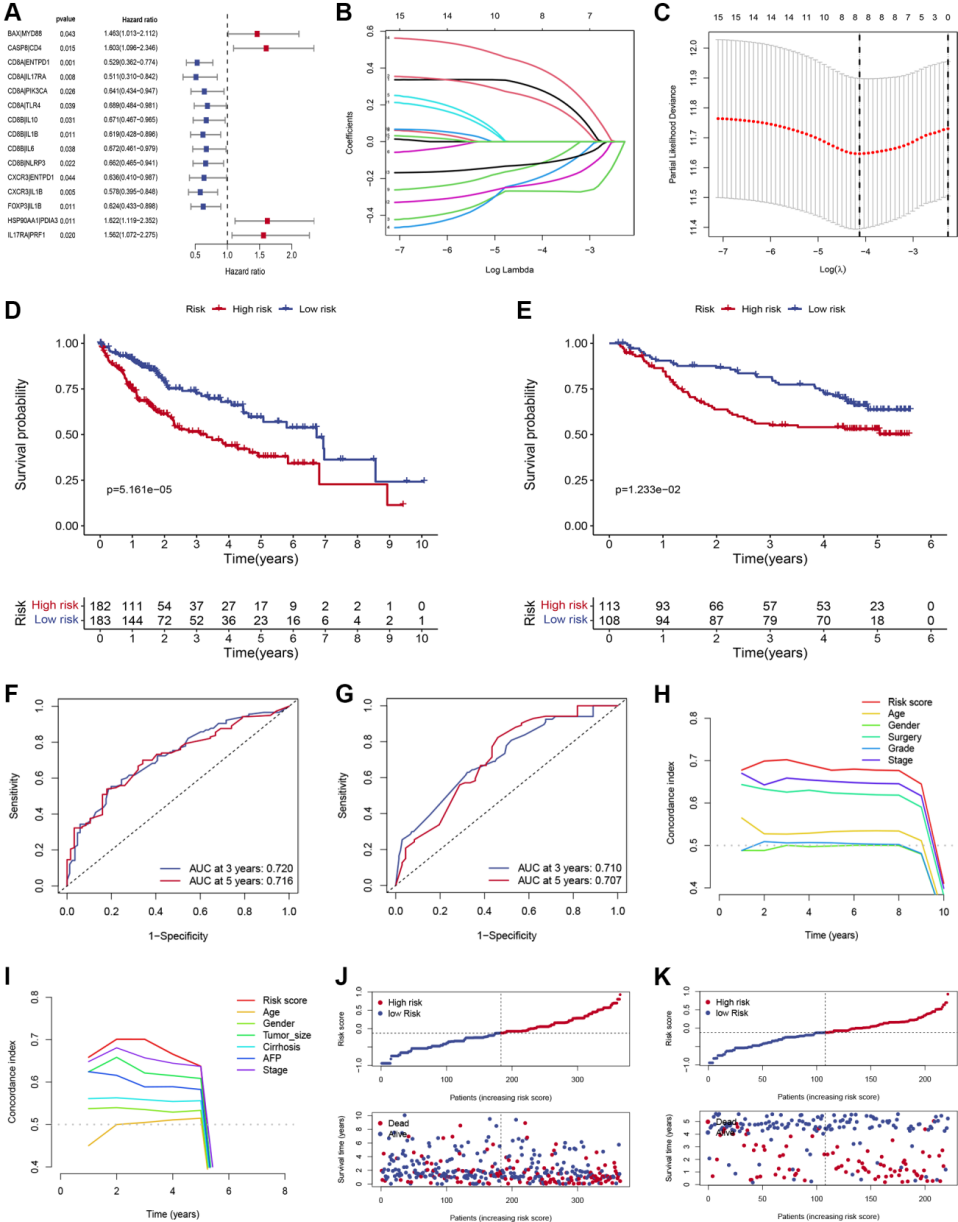
**Construction and verification of ICD-related prognostic gene signature.** (**A**) Forest plots showing the results of the Cox univariate regression of 15 ICD-related prognostic gene pairs. (**B**) The LASSO coefficient profile of 8 ICD-related prognostic gene pairs. (**C**) Selection of optimal LASSO model parameters for HCC patients (λ). (**D**, **E**) Kaplan-Meier survival curves of patients in low- and high-risk groups in training (**D**) and validation (**E**) sets. (**F**, **G**) The AUC values of the signature in the training (**F**) and validation sets (**G**). (**H**, **I**) C-index of the risk score and other clinical traits in the training (**H**) and validation sets (**I**). (**J**, **K**) Risk score and OS distributions were assessed in the training (**J**) and validation sets (**K**).

This study progressed according to the flow chart (Figure 1). A total of 89 ICD-related gene pairs were screened with the algorithm described in “Methods”. Of the 89 ICD-related gene pairs identified above, 15 were evidently associated with the OS of HCC patients in the training set (Figure 2A). These survival-associated ICD-related gene pairs were then used to generate a prognostic signature via a LASSO Cox regression approach (Figure 2B, 2C), ultimately identifying 8 gene pairs for inclusion in the developed signature. The coefficients of 8 ICD-related gene pairs were employed to calculate the risk score (Table 2). Its prognostic value was next assessed in the training set, with HCC patients being stratified into low- and high-risk groups. HCC patients with the high-risk score had a worse OS (*P* < 0.05; Figure 2D). The same formula and cut-off threshold were then applied to the validation set, which similarly revealed worse OS (Figure 2E). The AUCs of the training and validation sets were 0.720 and 0.710 at 3 years, 0.717 and 0.707 at 5 years, respectively, indicating that the signature has good predictive efficacy (Figure 2F, 2G). We then calculated the C-index of risk score and several clinical parameters. The highest C-index of the risk score affirmed the predictive utility of our signature in both training and validation sets (Figure 2H, 2I). Additionally, we compared the risk model with existing signatures in HCC. ROC curves indicated that our signature achieved significantly favorable predictive power compared with previously published prognostic models (Supplementary Figure 1). Risk score and OS distributions were shown in Figure 2J and it was noticed that the risk of death gradually increased with the increment of the risk score. The same analyses were then performed in the validation set and similar distribution was found (Figure 2K).

**Table 2:** The coefficients of 8 ICD-related gene pairs.

**ICD-related gene**	**Coefficient**
BAX|MYD88	0.2899
CASP8|CD4	0.2312
CD8A|ENTPD1	−0.2687
CD8A|IL17RA	−0.2006
CD8B|IL6	−0.1251
CXCR3|IL1B	−0.2297
FOXP3|IL1B	−0.1201
HSP90AA1|PDIA3	0.4057

### Development and validation of a nomogram

**Figure 3:**
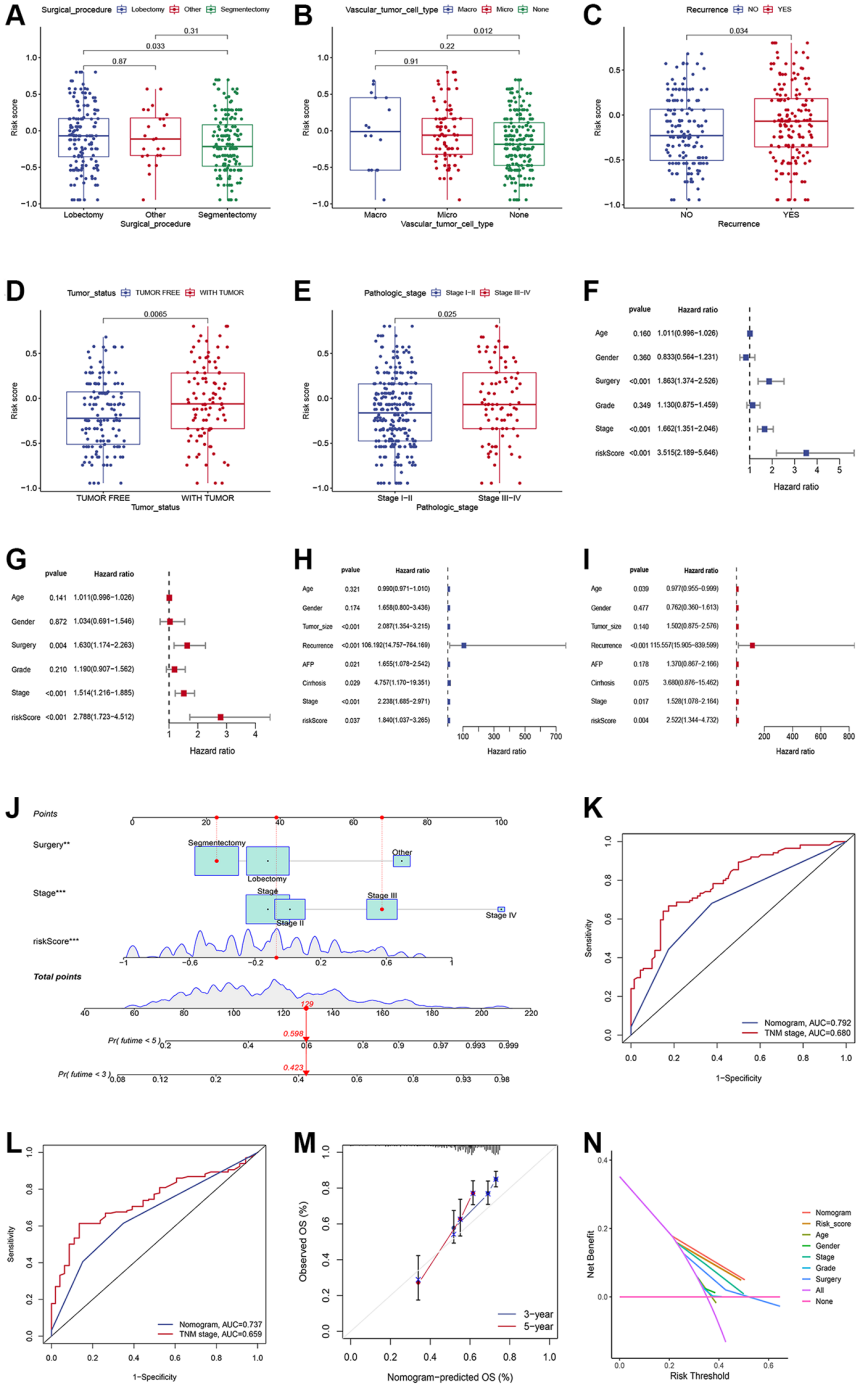
**Generation and validation of a nomogram scoring system.** (**A**–**E**) The relationship between the risk score, surgical procedure, vascular tumor cell type, recurrence status, tumor status, and stages. (**F**, **G**) Forest plots showing the results of the univariate (**F**) and multivariate (**G**) Cox analysis in the training set. (**H**, **I**) Forest plots showing the results of the univariate (**H**) and multivariate (**I**) Cox analysis in the validation set. (**J**) Nomogram predicting the 3-year and 5-year overall survival of HCC patients. (**K**, **L**) Comparison of the 3- (**K**) and 5-year AUC (**L**) of nomogram and TNM staging system. (**M**) The calibration plots of the nomogram at 3 and 5 years. (**N**) The DCA curves of the nomogram at 3 and 5 years.

We explored the relationship between the risk score and clinicopathological traits, including age, gender, surgical procedure, grade, vascular tumor cell type, recurrence status, tumor status, and stage. Significant correlations were observed between risk score and surgical procedure, vascular tumor cell type, recurrence status, tumor status, and stages (Figure 3A–3E). HCC patients who underwent segmentectomy had evidently higher risk scores than those who underwent lobectomy (Figure 3A). Significantly higher risk scores were observed in the Macro and Micro groups (Figure 3B). High-risk scores were more common in patients with recurrence status (Figure 3C) and with tumor status (Figure 3D). In addition, patients in stage pathological III-IV had higher risk scores than those in stage I-II (Figure 3E). Univariate and multivariable Cox regression analyses demonstrated the independent predictive roles of surgery, stage, and risk score in the training cohort (Figure 3F, 3G). The independent predictive roles of stage and risk score were further confirmed in the validation set (Figure 3H, 3I). To enhance the clinical use of the signature, risk score, surgery procedure, and stage were combined to build a prognostic nomogram (Figure 3J). The combined nomogram showed an excellent predictive ability and performed better than any TNM stage in predicting 3- and 5-year survival (Figure 3K, 3L). Calibration curves demonstrated that the nomogram performed well (Figure 3M). Meanwhile, the DCA curve demonstrated the contribution of risk and nomogram in clinical decision-making (Figure 3N).

### Assessment of immune landscape

**Figure 4:**
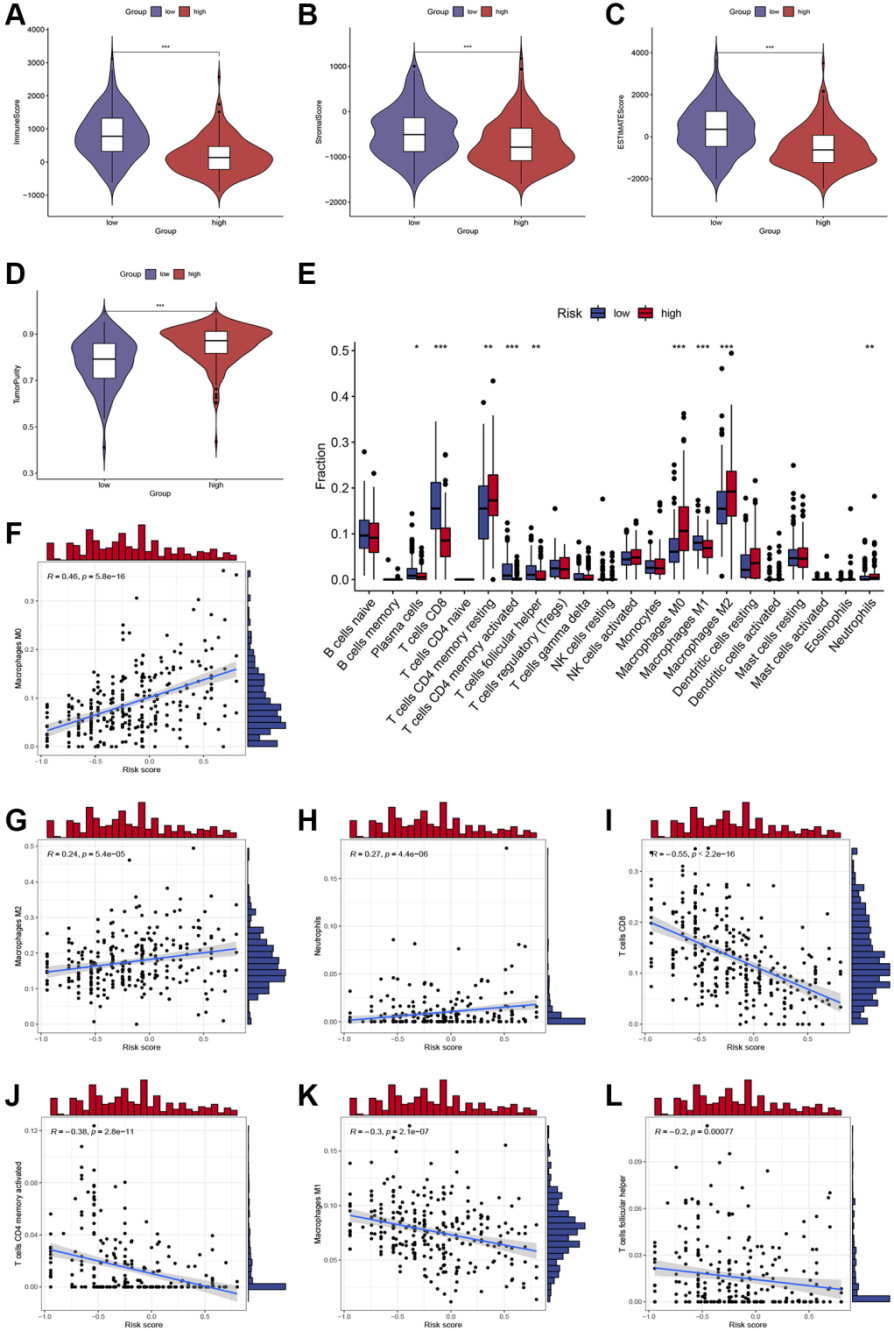
**Assessment of immune cell infiltration and the immune microenvironment in different subgroups.** (**A**–**D**) Differences in immune scores, stromal scores, and tumor purity between high-risk and low-risk groups. (**E**) Differences in immune cells infiltration between high-risk and low-risk groups. (**F**–**L**) Correlations between the risk score and immune cells infiltration. ^*^, ^**^, and ^***^ represent *p* < 0.05, 0.01, and 0.001, respectively.

The ESTIMATE algorithm was further used to process HCC patient sample data to compare the relative contribution of immune and stromal cells to the obtained patient samples. Compared with low-risk patients, patients with high-risk scores had significantly lower immune and stromal scores and higher tumor purity (*P* < 0.001; Figure 4A–4D). Based on the CIBERSORT algorithm, we observed increased CD8+ T cell, plasma cells, activated memory CD4+ T cell, M1 macrophage, and T follicular helper cell infiltration in low-risk patients, whereas high-risk patients exhibited enhanced resting memory CD4+ T cell, M0 macrophage, M2 macrophage, and neutrophils infiltration (*P* < 0.05; Figure 4E). The scatter plot (Figure 4F–4L) further showed that high-risk score was positively associated with several infiltrations of immune cells, such as M0 macrophage, M2 macrophage, and neutrophils (Figure 4F–4H), while the infiltration level of CD8+ T cell, activated memory CD4+ T cell, M1 macrophage, and Tfh cells was associated with the high-risk group (Figure 4I–4L).

### Immunotherapeutic response analyses and drug efficacy assessment

**Figure 5:**
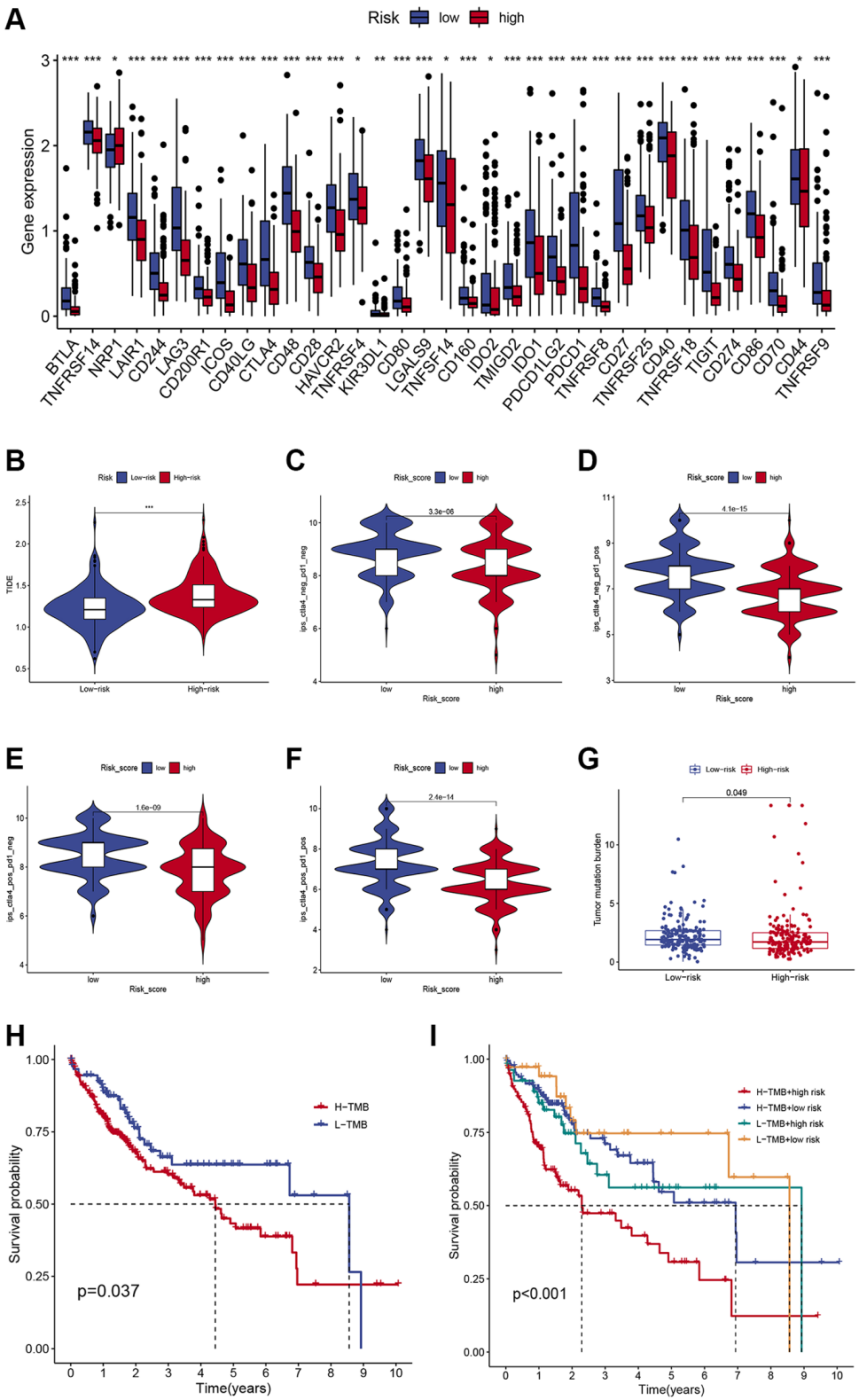
**Comparison of the immunotherapeutic response of low- and high-risk HCC patients.** (**A**) Comparison of immune checkpoint expression between the two subgroups. (**B**) Differences in TIDE scores in low- and high-risk individuals. (**C**–**F**) Differences in IPS scores in low- and high-risk individuals. (**G**) Differences in TMB in low- and high-risk individuals. (**H**) Kaplan-Meier survival curves of HCC patients in different TMB groups. (**I**) Kaplan-Meier survival curves in different risk scores and TMB subgroups.

We compared the expression of major immune checkpoints including HAVCR2, PD-1, and PD-L1, between the two subgroups. Overall, most of the immune checkpoints were substantially elevated in the low-risk group (Figure 5A), indicating that patients with low-risk scores may achieve better ICIs therapy results. Moreover, we use the TIDE algorithm to evaluate the likelihood of HCC benefiting from ICI therapy. The results demonstrated that the TIDE score was higher in high-risk patients (Figure 5B), suggesting tumors in high-risk patients could acquire immune escape more easily. Furthermore, we compared the immunotherapy efficacy of the two risk subgroups by IPS scores and found that in the anti-CTLA4 single-drug group, the anti-PD1 single-drug group, and the anti-CTLA4 and anti-PD1 combination group, the IPS scores were higher in the low-risk group (Figure 5C–5F), suggesting that patients with low-risk HCC may have greater sensitivity to immunotherapy. Growing evidence suggests that TMB may determine the individual response to cancer immunotherapy. Correlation analysis demonstrated that the risk scores were negatively associated with TMB (Figure 5G), indicating low-risk patients may respond better to immunotherapy. In addition, K-M survival analysis demonstrated that low-TMB patients enjoyed a much longer survival time than their counterparts (Figure 5H). Combining TMB and risk score allowed us to classify patients into four groups. The combined survival analysis showed that the high TMB and high- risk groups had the worst prognoses, and, conversely, the low TMB and low-risk groups had the best prognoses (Figure 5I).

**Figure 6:**
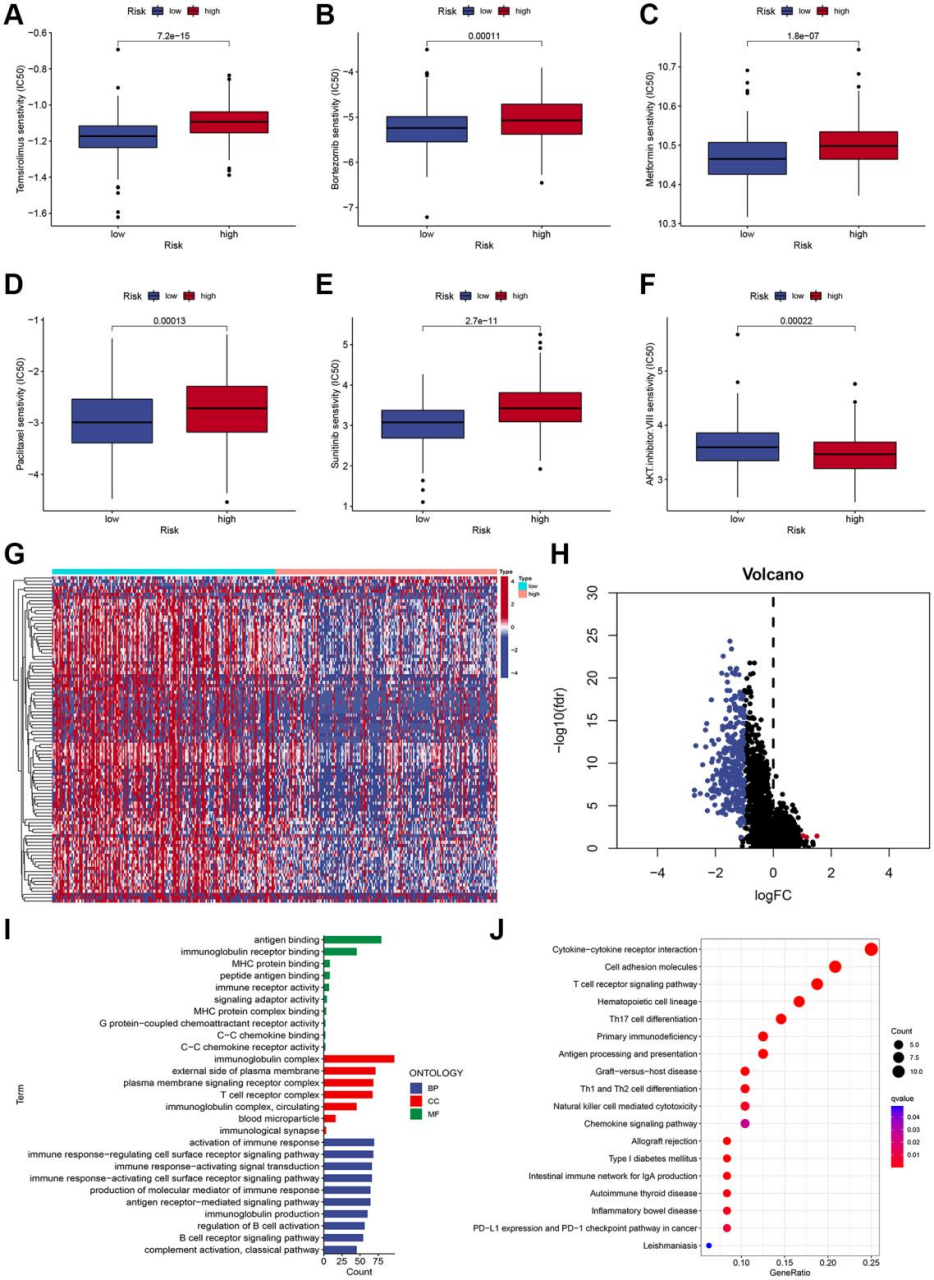
**Chemotherapy sensitivity and functional enrichment analyses.** (**A**–**F**) Boxplots of the IC50 values of the Temsirolimus, Bortezomib, Metformin, Paclitaxel, Sunitinib, and AKT inhibitor VIII between different risk subgroups. (**G**, **H**) Heatmap (**G**) and volcano plots (**H**) demonstrate DEGs between two risk subgroups. (**I**) GO terms of DEGs among different risk subgroups. (**J**) KEGG enrichment analyses of DEGs among different risk subgroups.

To identify a drug therapy target, a crucial way is to clarify the correlation between ICD-related signatures and antitumor drugs. As shown in Figure 6, the IC50 values of Temsirolimus, Bortezomib, Metformin, Paclitaxel, and Sunitinib were higher in the high-risk subgroup (Figure 6A–6E), while the IC50 value of AKT inhibitor VIII was higher in the low-risk subgroup (Figure 6F).

### GO and KEGG enrichment analyses

To characterize the functional enrichment in risk groups, we compared the mRNA expression difference between two risk subgroups to identify DEGs (|log2 (fold change, FC) | > 1 and adjusted *P* < 0.05), and a total of 314 DEGs were identified (Figure 6G, 6H). As shown in Figure 6I, GO terms indicated that these DEGs were mainly correlated with antigen binding, MHC protein binding, and immunoglobulin receptor binding. Additionally, KEGG pathway analysis demonstrated that these DEGs were mainly associated with immune-related pathways, including the T cell receptor signaling pathway, Cytokine-cytokine receptor interaction, Cell adhesion molecules, and Th17 cell differentiation (Figure 6J).

## DISCUSSION

The past decade has witnessed breakthroughs in cancer immunotherapy, which has been incorporated into the treatment regimen of various tumors, but its response rate remains low due to immune evasion of cancer cells and tumor resistance to conventional therapies. ICD is considered to be one of the most promising approaches to achieving the complete elimination of tumor cells, as it can activate T-cell adaptive immune responses and lead to the formation of long-term immune memory [14]. In addition, hundreds of studies have shown that after induction chemotherapy with ICDs, tumors change from “cold” to “hot” in response to immune checkpoint inhibitors, indicating that ICDs will open the door to immunotherapy [15–17]. Currently, two ICD-related anticancer drugs have come out. One is belantamab mafodotin, approved by the FDA in 2020 to treat adult patients with relapsed or refractory multiple myeloma [18]; the other is the FDA-approved lurbinectedin for the treatment of small cell lung cancer [19]. Hence, given that ICD-associated biomarkers benefit from immunotherapy, identifying these biomarkers may help differentiate HCC patients.

Our study comprehensively analyzed the expression profiles of these ICD-related genes and established a risk signature with 8 ICD-related gene pairs through a series of computational methods. Risk scores established using the model allowed the grouping of HCC patients into two distinct risk-based populations with significantly different OS, and this model yielded a high AUC value and C-index. And it was successfully externally validated in the GEO cohort, indicating a better capability. Some of the ICD-related genes included within the established signature have previously been shown to be correlated with HCC tumorigenesis. FOXP3, for example, can inhibit tumor growth and induced apoptosis in HCC by targeting c-Myc [20]. Correlation analysis demonstrated that risk score was closely associated with surgical procedure, vascular tumor cell type, recurrence status, tumor status, and stages. Multivariate Cox analysis revealed the independent prognostic role of the prognostic signature. To enhance the clinical use of the ICD-relate gene signature, risk score, surgery procedure, and stage were combined to build a nomogram, and the combined nomogram showed outstanding predictive ability. Moreover, the AUC of the nomogram developed herein was superior to that of the TNM stage, emphasizing its robust predictive utility.

Hundreds of studies have shown the tumor microenvironment (TME) to be a key determinant of tumorigenesis and disease progression, as tumor-associated cells can shape important malignant processes. Immune cells, as major components of the tumor microenvironment, determines the survival and response to immunotherapy. For example, clinical data strongly suggest that the immune cell composition of tumors in HCC affects treatment response and is strongly associated with patient outcomes [21–23]. Our study showed that patients in the low-risk group presented with more pronounced immune cell infiltration relative to high-risk individuals. Specifically, increased CD8+ T cell, plasma cells, activated memory CD4+ T cell, M1 macrophage, and Tfh cell infiltration were observed in low-risk patients, whereas high-risk patients exhibited enhanced resting memory CD4+ T cell, M0 macrophage, M2 macrophage, and neutrophils infiltration. Tumor-infiltrating lymphocyte populations are closely linked with HCC patient outcomes, and T cells are the best-studied lymphocyte type [24]. T cells are one of the most common immune cells found in HCC tumor tissues, and they can mediate the protection of tumor cells, but are often dysfunctional and depleted in cancer [25, 26]. Barsch et al. [26] found that HCC patients dominated by depleted CD8+ T cells had poor OS and progression-free survival. Consistently, the present study demonstrated that CD8+ T cell infiltration was decreased in high-risk patients. Tumor-associated macrophages (TAM) are predominantly M2 macrophages, and high-density TAM infiltration in HCC is a marker of poor prognosis [27, 28]. M2 macrophages drive tumor growth directly and indirectly by suppressing cytotoxic cell populations, including NK cells and CD8+ T cells [27]. M2 macrophages can upregulate PDL1 expression in HCC, thereby suppressing CD8+ T cell activity [29, 30]. Neutrophils were also reported to suppress T-cell immunity and promote tumor progression [31]. Moreover, we also observed that patients with high-risk scores had evidently lower TME scores and higher tumor purity compared to low-risk patients.

Immunotherapy based on ICIs has become a powerful clinical strategy for treating HCC [3, 32]. Currently, atezolizumab in combination with bevacizumab is approved for third-line treatment of advanced HCC [33]. In this work, most immune checkpoints were evidently elevated in the low-risk group, suggesting that low-risk HCC patients may respond more readily to immunotherapy which can provide a reference for clinical drug selection of ICIs. Moreover, a higher TIDE score was observed in high-risk patients, suggesting tumors in high-risk patients could acquire immune escape more easily. The IPS results demonstrated that the immunotherapy response was poorer in the high-risk group, which was in accordance with the previous results. In addition, several biomarkers, notably PD-L1, TMB, and other biomarkers, have been shown to have significant predictive value in HCC immunotherapy [34]. Growing evidence suggests that high TMB was associated with better response to immunotherapy. In this study, correlation analysis revealed that the risk scores were negatively associated with TMB, indicating low-risk patients may have a better response to immunotherapy.

Surgery followed by adjuvant treatment is the most common treatment for HCC patients. Chemotherapy and targeted therapy are one of the most important treatment modalities for advanced HCC [35, 36]. We explored the correlation between the risk score and antitumor drugs. The results revealed the IC50 values of Temsirolimus, Bortezomib, Metformin, Paclitaxel, and Sunitinib were higher in the high-risk group, while the IC50 value of AKT inhibitor VIII was higher in the low-risk group. These data indicated that the risk signature might predict potential response to immunotherapy, chemotherapy, or targeted therapy.

Indeed, some limitations could be found in this study. First, these analyses were retrospective in design and necessitate future prospective validation. In addition, HCC tissues and cell lines are required to verify the expression of signature genes, and more functional assays are needed to verify the roles of signature genes in the future. Second, while gene expression data were used to gauge the intratumoral immune cell landscape within HCC patient tumors, these analyses require cell-based validation. Moreover, the presence of cells within tumors does not necessarily indicate that these cells interact with one another, highlighting a need for detailed research efforts aimed at validating all aspects of this study.

## CONCLUSIONS

The study developed and validated an effective risk model based on 8 ICD-related gene pairs in HCC. Our research provides many useful insights for predicting the prognosis of HCC patients and provides novel insight into the potential therapeutic strategy for HCC patients.

## Supplementary Materials

Supplementary Figure 1

Supplementary Table 1
