# Supplementary material for: Establishing a signature based on immunogenic cell death-related gene pairs to predict immunotherapy and survival outcomes of patients with hepatocellular carcinoma
Source: Aging (Albany NY). 2022 Dec 14;14(23):9699–714. doi: 10.18632/aging.204419 (PMC9792212; doi:10.18632/aging.204419)
Supplement: Supplementary Figure 1 [file aging-14-204419-s001.pdf]

SUPPLEMENTARY FIGURE

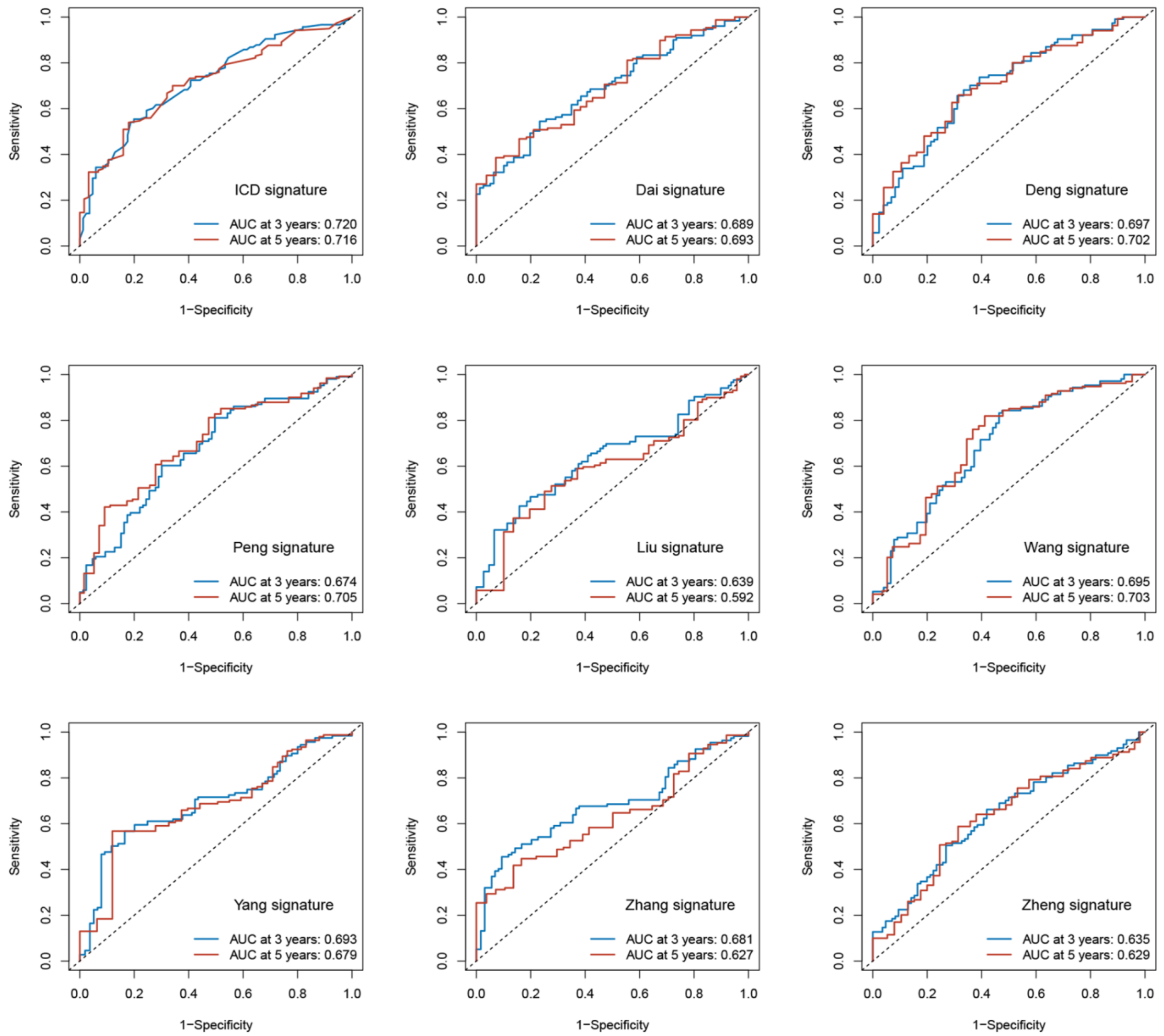

Supplementary Figure 1. Time-dependent ROC curves of prognostic risk model and other prognostic models.
