# Supplementary material for: Establishing a signature based on immunogenic cell death-related gene pairs to predict immunotherapy and survival outcomes of patients with hepatocellular carcinoma
Source: Aging (Albany NY). 2022 Dec 14;14(23):9699–714. doi: 10.18632/aging.204419 (PMC9792212; doi:10.18632/aging.204419)
Supplement: Supplementary Table 1 [file aging-14-204419-s002.pdf]

SUPPLEMENTARY TABLE

Supplementary Table 1. The list of 34 ICD-related genes.

ATG5	EIF2AK3	IL10	NT5E
BAX	ENTPD1	IL17A	P2RX7
CALR	FOXP3	IL17RA	PDIA3
CASP1	HMGB1	IL1B	PIK3CA
CASP8	HSP90AA1	IL1R1	PRF1
CD4	IFNA1	IL6	TLR4
CD8A	IFNB1	LY96	TNF
CD8B	IFNG	MYD88	
CXCR3	IFNGR1	NLRP3	
